# The association between urinary cotinine level and metabolic syndrome profiles among adolescents: findings from the Ewha Birth and growth study

**DOI:** 10.1186/s12889-023-15458-5

**Published:** 2023-04-21

**Authors:** Hyunjin Park, Ui-Jeong Kim, Eun Jeong Choi, Seunghee Jun, Bomi Park, Hye Ah Lee, Hae Soon Kim, Hyesook Park

**Affiliations:** 1 Department of Preventive Medicine, College of Medicine, Ewha Womans University, 25 Magokdong-ro 2-gil, Gangseo-gu, Seoul, 07804 Republic of Korea; 2 Graduate Program in System Health Science and Engineering, Ewha Womans University, Seoul, Republic of Korea; 3 Department of Preventive Medicine, College of Medicine, Chung-Ang University, Seoul, Republic of Korea; 4 Clinical Trial Center, Mokdong Hospital, Ewha Womans University, Seoul, Republic of Korea; 5 Department of Pediatrics, College of Medicine, Ewha Womans University, Seoul, Republic of Korea

**Keywords:** Cotinine, Secondhand smoke, Metabolic syndrome, Adolescents, Cohort

## Abstract

**Background:**

Secondhand smoke (SHS) exposure among adolescents who are still developing can negatively affect their physical and psychological health, including metabolic syndrome (MetS), which is a risk factor for cardiovascular disease. However, the relationship between exposure to SHS and MetS in adolescence has not been evaluated.

**Methods:**

A total of 240 subjects aged 13–15 years who were followed up in the Ewha Birth and Growth Study were included in this study. Using the urinary cotinine level, the participants’ exposure to SHS was divided into tertiles, and the continuous MetS score (cMetS) and its components were compared among the three groups using a generalized linear model and trend analysis. Univariate and multivariate linear regression analyses were performed. We adjusted for several confounding variables including sex, father’s education level, father’s current alcohol consumption status, moderate physical activity, and overweight status.

**Results:**

The association between cMetS and the urinary cotinine level was not significant. However, the higher the urinary cotinine level, the lower the high-density lipoprotein cholesterol (HDL-C) level. In particular, the significance of the HDL-C level was maintained after adjusting for covariates.

**Conclusions:**

This study supports an association between SHS exposure and the components of MetS in adolescents aged 13–15 years, and it suggests the need to address SHS exposure in adolescents to reduce the cardiovascular risk in later life.

## Introduction

Globally, approximately one-third of adults are exposed to secondhand smoke (SHS) [1], resulting in the deaths of an estimated 600,000 non-smokers each year [2]. Exposure to SHS remains common despite evidence of negative health effects in adolescents. According to a CDC report, more than a third of non-smokers aged 12–17 years in the United States [3] and 48% of adolescents aged 12–19 years in Canada [4] have been exposed to SHS. According to the results of the Korea Youth Risk Behavior Web-Based Survey (KYRBS) in 2020, the rate of exposure to SHS at home is 25.4%, and that to indoor SHS in public places is 42.2% [5].

Adolescence is the transitional period from childhood to adulthood. It is an important period that affects one’s health status in adulthood, and SHS exposure during this period has a harmful effect on health. It has the potential to cause asthma, respiratory disease, hypertension, and chronic kidney disease [6–8], and it negatively affects mental health, including depression and suicide [9]. It also affects academic achievement and neurocognitive performance and increases the risk of smoking among adolescents [10, 11]. A study of 12–19-year-old American adolescents reported that increased exposure to SHS during adolescence resulted in a 5.4% higher prevalence of metabolic syndrome (MetS) [12]. However, few studies have evaluated the association between MetS and its components by examining the urinary cotinine level in adolescents.

MetS is a disease consisting of at least three of the following five conditions: abdominal obesity, high blood pressure (BP), high blood glucose and triglyceride (TG) levels, and a low high-density lipoprotein cholesterol (HDL-C) level. MetS is associated with an increased cardiovascular disease risk [13]. A recent study of Korean adolescents on the prevalence of MetS and changes in risk factors reported that the prevalence has increased from 1.7 to 2.2% over the past 12 years (2007–2018) [14]. MetS in adolescence often continues into adulthood, leading to cardiovascular disease and type 2 diabetes [15, 16]. In a previous study, after categorizing the risk for cardiovascular disease in 13-year-old adolescents, when observed 4 years later, more than 12% were in the high-risk group [17]. This suggests that interventions targeting MetS in early adolescence are necessary to reduce cardiovascular disease in later life.

We evaluated the level of exposure to SHS and its influence on metabolic markers in 13–15-year-old adolescents who participated in the Ewha Birth and Growth Study by measuring the urinary cotinine level, a biomarker of SHS.

## Materials and methods

### Study subjects

This study used data from the Ewha Birth and Growth Study. This study was conducted in women who visited Ewha Womans University Mokdong Hospital for prenatal examinations at 24–28 weeks of pregnancy during 2001–2006 and who agreed to participate in the cohort study. Among them, 940 children born to pregnant women with no medical history before pregnancy and who had consented to participate in the study have been followed up since 2005. Follow-up was conducted at 3 years of age, 5 years of age, and from 7 up to 13 years of age. Detailed information on the cohort has been published elsewhere [18]. The follow-up survey of 13-year-olds was conducted from 2015, and we used data collected at the age of 13 in this study. If the subjects did not participate in the 13-year-old follow-up examination, the data collected at the age of 14 or 15 were used. Therefore, data from a total of 248 adolescents (121 boys and 127 girls) were collected at follow-up at the age of 13 to 15 years. In all, 240 subjects (13 years old, n = 188; 14 years old, n = 33; 15 years old, n = 19) were ultimately included in the analysis, excluding subjects who did not have data on the metabolic index of MetS components and the measurement of urinary levels of cotinine.

### Measurement of urinary cotinine levels

During follow-up between 13 and 15 years of age, fasting urine samples were collected on the day of the examination and dispensed into 20 mL sterile storage containers. All urine samples were stored at − 80 °C until analysis. Urinary cotinine was measured using a high-performance liquid chromatography-triple tandem mass detector (Q-sight 210, PerkinElmer, Waltham, MA, USA) [19]. The limit of detection (LOD) of cotinine was 0.149 µg/L. Of the 240 subjects in this study, the cotinine level was below the LOD in 18 (7.5%) and thus was assigned the value of the square root of the LOD in the analysis [20]. Participants’ exposure to SHS was divided by urinary cotinine level by tertile (1st tertile; ≤ 0.356 ug/L, 2nd tertile; 0.357–0.692 ug/L, 3rd tertile; > 0.692 ug/L).

### Definition of continuous metabolic syndrome score

MetS in children and adolescents does not have a universal and uniform definition compared to adults [21]. For obesity, MetS is defined by considering body mass index (BMI) or waist circumference (WC). It should be taken into account that the cutoffs for BMI and WC may differ by sex, age, and race [22] and that there is no consensus on the optimal measurement method [23]. MetS severity using BMI has much greater clinical potential than the measure using WC [22]. Therefore, we defined MetS according to the WHO guidelines [24] using BMI.

Most studies use the adult definition, with modified cut-off points for each component [25]. To overcome these limitations, the cMetS has been proposed, which is a more robust measure for MetS than previous categorical measures as a continuous variable [26, 27]. Recently, the usefulness of cMetS in childhood epidemiological studies has increased. When evaluating the validity of cMetS in subjects aged 7–18 years (average age 12.5 years), the mean cMetS increased as the number of MetS components increased. This was accurate and sensitive enough to predict MetS (area under the curve = 0.94) [28]. In addition, childhood MetS, defined as cMetS, is related to cardiovascular risk in early adulthood [29]. Therefore, in this study, cMetS was used to determine the MetS status of adolescents.

Additionally, WC and BMI are used to estimate obesity when assessing cMetS. Because there was no significant difference between cMetS based on WC and cMetS based on BMI, cMetS was calculated using BMI in the study [30].

Therefore, we calculated cMetS based on BMI, fasting blood glucose (FBG), TG levels, mean arterial pressure (MAP), and HDL-C. BMI was calculated as body weight (kg) divided by height squared (m^2^). For these measurements, an automatic height scale (GL-150, G-Tech International Co., Ltd., Uijeongbu, South Korea) was used, with participants wearing light clothing and no shoes. BP was measured using an automatic blood pressure monitor (BPBIO320, InBody Co., Ltd., Seoul, South Korea) when the subject was in a stable state. The MAP was calculated using the formula (diastolic BP + [systolic BP – diastolic BP]) ÷ 3 [31], which yields a small standard deviation compared with systolic and diastolic BP, making it easy to calculate the cMetS. At the time of follow-up, blood tests were performed using blood collected from the health checkup, to obtain MetS values, including TG, HDL-C, and FBG levels. All anthropometric and BP measurements were performed by trained researchers or nurses.

To generate cMetS, standardized values were calculated for the MetS components (BMI, FBG, TG, MAP, and HDL-C) using the Z-score method. Because HDL-C has an inverse association, the standardized value of HDL-C was multiplied by − 1. The cMetS was calculated using the formula (BMI + FBG + TG + MAP – HDL-C), with a higher value predicting a relatively higher risk of MetS [28].

### Covariates

Through a literature review [12, 32, 33], sex, father’s education level, father’s current alcohol consumption status, moderate physical activity frequency, being overweight, and obesity were judged as potential confounding factors. Father’s education level was redefined from the existing questionnaire items (elementary and middle school graduation, high school graduation, university graduation, graduate school graduation, or higher) into two categories for analysis: high school graduation of lower and university and graduate school graduation or higher. Based on the sex- and age-specific BMI percentile data from the 2017 Korean National Growth Charts for children and adolescents [34], the normal (< 85th percentile) and overweight/obesity groups (≥ 85th percentile) were defined.

### Statistical analyses

First, the normal distribution of MetS-related components was evaluated. For continuous variables in the descriptive analysis, the results are presented as the mean ± standard deviation. Because the TG level did not follow a normal distribution it was log transformed for the analysis. The mean differences in the cMetS and MetS components according to basic characteristics were evaluated using the t-test and generalized linear model.

To evaluate the association between the metabolic indicators according to the urinary cotinine level, the average difference was determined by dividing the cotinine level into tertiles using a generalized linear model. In addition, a trend analysis was performed on the mean change according to exposure level. The cotinine level was considered an ordinal variable, and univariate linear regression analysis was performed to evaluate the associations of the metabolic syndrome score and MetS components with the urinary cotinine level. In addition, multivariate linear regression analysis was conducted considering environmental factors such as parents’ social level and health behavior as well as individual-level factors such as obesity status and health behavior of the subjects. Thus, model 1 was adjusted for sex and environmental factors (father’s education level and father’s current drinking), and model 2 was analyzed by additionally adjusting for individual-level factors (being overweight and engaging in moderate physical activity) including model 1. All statistical analyses were performed using SAS program version 9.4 (SAS Institute, Cary, NC, USA), and statistical significance was evaluated based on a significance level of 0.05 using a two-sided test.

### Ethics statement

The parents or guardians of all participants provided written informed consent, and the study protocol was approved by the Institutional Review Board (IRB) of Ewha Womans University Seoul Hospital (number: SEUMC 2020-07-016-002).

## Results

Among 240 subjects, almost half were boys (n = 119, 49.6%), and the average age was 13.30 ± 0.61 years. cMetS was significantly associated with overweight status (p < 0.001). In addition, the overweight and obesity group showed a significant relationship with MAP, systolic and diastolic BP, and TG level. The average systolic BP was 110.79 ± 11.90 mmHg, and there was a significant difference according to sex (112.7 mmHg in boys and 108.9 mmHg in girls, *p* = 0.01) (Table 1).

**Table 1:** Distribution of metabolic syndrome components to the basic characteristics of study subjects

Variables	cMetS	MAP (mmHg)	SBP (mmHg)	DBP (mmHg)	BMI (kg/m^2^)	FBG (mg/dL)	log TG^a^	HDL-C (mg/dL)
**Total**	0.00 ± 3.03	81.89 ± 8.93	110.79 ± 11.90	67.43 ± 8.74	20.75 ± 3.39	93.21 ± 6.42	4.23 ± 0.44	51.36 ± 9.69
Boys (n = 119)	0.00 ± 3.13	82.96 ± 9.49	112.69 ± 12.30	68.10 ± 9.53	21.08 ± 3.83	93.38 ± 5.89	4.19 ± 0.47	51.16 ± 9.01
Girls (n = 121)	0.00 ± 2.93	80.83 ± 8.26	108.92 ± 11.23	66.78 ± 7.87	20.42 ± 2.88	93.04 ± 6.91	4.28 ± 0.41	51.56 ± 10.34
*P* value	1.000	0.064	0.014	0.242	0.130	0.685	0.098	0.748
**Urinary cotinine level** ^**b**^ **(ug/L)**								
T1 (≤ 0.356 ug/L) (n = 80)	-0.54 ± 2.98	81.43 ± 8.29	110.66 ± 11.74	66.81 ± 8.36	20.37 ± 3.26	92.66 ± 5.52	4.20 ± 0.46	53.75 ± 10.76
T2 (0.357–0.692 ug/L) (n = 79)	0.18 ± 2.84	81.61 ± 8.57	109.53 ± 10.95	67.65 ± 8.33	20.41 ± 3.01	94.08 ± 7.07	4.31 ± 0.42	50.82 ± 9.58
T3 (≥ 0.693 ug/L) (n = 81)	0.36 ± 3.21	82.60 ± 9.91	112.15 ± 12.90	67.83 ± 9.54	21.45 ± 3.78	92.90 ± 6.56	4.20 ± 0.44	49.53 ± 8.20
*P* value	0.142	0.669	0.379	0.734	0.072	0.333	0.171	0.018
**Secondhand smoking** ^**c**^								
No (n = 217)	-0.05 ± 2.96	81.60 ± 8.88	110.38 ± 11.60	67.21 ± 8.83	20.70 ± 3.32	93.32 ± 6.50	4.24 ± 0.44	51.48 ± 9.70
Yes (n = 20)	0.69 ± 3.72	85.31 ± 9.49	115.38 ± 14.96	70.28 ± 7.79	21.31 ± 4.15	92.45 ± 5.79	4.28 ± 0.42	50.30 ± 9.99
*P* value	0.302	0.077	0.074	0.135	0.439	0.565	0.655	0.604
**Overweight**								
Normal (BMI < 85th) (n = 183)	-0.67 ± 2.38	81.09 ± 8.60	109.4 ± 11.21	66.94 ± 8.53	-	92.91 ± 5.88	4.19 ± 0.40	52.43 ± 9.69
Overweight (BMI ≥ 85th) (n = 57)	3.81 ± 3.47	86.37 ± 9.60	118.64 ± 12.76	70.24 ± 9.54	-	94.89 ± 8.80	4.5 ± 0.57	45.31 ± 7.21
*P* value	< 0.001	0.001	< 0.001	0.037	-	0.088	< 0.001	< 0.001
**Moderate** **physical activity**								
Never (n = 46)	0.49 ± 3.50	81.40 ± 9.76	109.77 ± 13.02	67.22 ± 8.80	21.55 ± 3.93	92.48 ± 6.35	4.29 ± 0.41	48.41 ± 9.80
1 ~ 2 times/week (n = 102)	0.09 ± 2.95	82.39 ± 8.23	112.16 ± 11.21	67.50 ± 8.40	20.63 ± 3.38	94.21 ± 7.16	4.22 ± 0.43	52.12 ± 9.89
3 ~ 4 times/week (n = 65)	-0.17 ± 2.79	82.48 ± 9.43	110.73 ± 11.85	68.35 ± 9.18	20.42 ± 3.05	92.18 ± 5.49	4.27 ± 0.48	51.94 ± 9.89
≥ 5 times/week (n = 23)	-0.79 ± 3.22	78.51 ± 8.73	107.13 ± 12.94	64.20 ± 8.72	21.10 ± 3.25	92.22 ± 4.58	4.07 ± 0.47	51.39 ± 7.02
*P* value	0.390	0.261	0.281	0.275	0.331	0.154	0.238	0.165
**Father’s drink experience** **(in the past year)**								
Never (n = 23)	0.58 ± 3.10	84.26 ± 9.00	111.65 ± 13.35	70.57 ± 7.56	21.26 ± 3.20	93.22 ± 5.10	4.32 ± 0.41	51.17 ± 9.80
≤ 1 times/week (n = 115)	-0.23 ± 2.77	81.96 ± 8.30	111.59 ± 10.82	67.14 ± 8.58	20.56 ± 3.40	92.95 ± 7.28	4.22 ± 0.41	51.87 ± 9.46
2 ~ 3 times/week (n = 67)	0.30 ± 3.48	81.92 ± 9.95	110.28 ± 13.19	67.75 ± 9.45	21.00 ± 3.67	93.37 ± 5.23	4.26 ± 0.50	50.91 ± 10.69
≥ 4 times/week (n = 32)	-0.21 ± 2.91	79.44 ± 8.89	107.88 ± 12.06	65.22 ± 8.45	20.68 ± 2.98	93.31 ± 6.47	4.17 ± 0.46	50.28 ± 7.50
*P* value	0.514	0.262	0.449	0.159	0.745	0.975	0.590	0.832
**Father’s education level** ^**d**^								
Low (n = 32)	0.66 ± 3.42	82.84 ± 11.41	112.30 ± 15.01	68.11 ± 11.41	21.25 ± 3.60	94.03 ± 4.87	4.33 ± 0.46	51.06 ± 8.00
High (n = 203)	-0.11 ± 2.97	81.71 ± 8.57	110.57 ± 11.44	67.29 ± 8.35	20.69 ± 3.38	93.03 ± 6.63	4.22 ± 0.44	51.40 ± 9.83
*P* value	0.187	0.511	0.448	0.624	0.396	0.413	0.169	0.852

cMetS, continuous metabolic syndrome score; MAP, mean arterial pressure; SBP, systolic blood pressure; DBP, diastolic blood pressure; BMI, body mass index; FBG, fasting blood glucose; TG, triglyceride; HDL-C, high-density lipoprotein-cholesterol.

^a^ Log transformation was performed because it was not normally distributed.

^b^ The range of urinary cotinine levels was divided into 1st tertile (≤ 0.356 ug/L), 2nd tertile (0.357–0.692 ug/L), 3rd tertile (≥ 0.693 ug/L).

^c^ Whether the child has been exposed to secondhand smoke in the home (self-reported).

^d^ High school graduation of lower and university and graduate school graduation or higher.

Table 2 shows the average differences in the cMetS and MetS components according to the cotinine level tertile. The mean cMetS was − 0.54 in the first tertile, 0.18 in the second tertile, and 0.36 in the highest tertile of the cotinine level, but there was no significant difference among the tertiles. The average BMI was 20.37 kg/m^2^ in the first tertile of the cotinine level, 20.41 kg/m^2^ in the second tertile, and 21.45 kg/m^2^ in the third tertile. BMI increased significantly from the lowest to highest cotinine level tertile (*p* for trend = 0.04). The mean HDL-C level was 53.75 mg/dL in the first tertile, 50.82 mg/dL in the second tertile, and 49.53 mg/dL in the third tertile. The difference among the groups and the trend analysis were significant. There was no significant difference in the other metabolic components according to the urinary cotinine level.

**Table 2:** Mean differences in metabolic syndrome components by urinary cotinine tertiles

Variable	Means (95% CI)			*P* value	*P* for trend
	T1 (n = 80)	T2 (n = 79)	T3 (n = 81)		
cMetS	-0.54 (-1.20, 0.13)	0.18 (-0.49, 0.85)	0.36 (-0.30, 1.02)	0.142	0.062
MAP (mmHg)	81.43 (79.45, 83.40)	81.61 (79.63, 83.60)	82.60 (80.64, 84.57)	0.669	0.403
SBP (mmHg)	110.66 (108.04, 113.28)	109.53 (106.89, 112.17)	112.15 (109.54, 114.75)	0.379	0.425
DBP (mmHg)	66.81 (64.88, 68.74)	67.65 (65.71, 69.60)	67.83 (65.91, 69.75)	0.734	0.461
BMI(kg/m^2^)	20.37 (19.63, 21.11)	20.41 (19.66, 21.15)	21.45 (20.71, 22.19)	0.072	0.043
FBG (mg/dL)	92.66 (91.25, 94.08)	94.08 (92.65, 95.50)	92.90 (91.50, 94.31)	0.333	0.818
log TG^a^	4.20 (4.10, 4.29)	4.31 (4.21, 4.41)	4.20 (4.10, 4.29)	0.171	0.983
HDL-C (mg/dL)	53.75 (51.64, 55.86)	50.82 (48.7, 52.94)	49.53 (47.44, 51.62)	0.018	0.006

The range of urinary cotinine levels was divided into 1st tertile (≤ 0.356 ug/L), 2nd tertile (0.357–0.692 ug/L), 3rd tertile (> 0.692 ug/L).

95% CI, 95% confidence intervals; cMetS, continuous metabolic syndrome score; MAP, mean arterial pressure; SBP, systolic blood pressure; DBP, diastolic blood pressure; BMI, body mass index; FBG, fasting blood glucose; TG, triglyceride; HDL-C, high-density lipoprotein-cholesterol.

^a^ Log transformation was performed because it was not normally distributed.

Table 3 shows the results of the linear regression analysis evaluating the associations between the cMetS and MetS components according to the cotinine level in urine. BMI increased by 0.54 kg/m^2^ (*p* = 0.04), and the HDL-C level decreased by − 2.11 mg/dL (*p* = 0.01), with increasing cotinine level tertile. However, after adjusting for the parents’ socioeconomic level, BMI no longer showed a significant relationship with the cotinine level, but the HDL-c level decreased significantly, by − 1.93 mg/dL, as the cotinine level tertile increased (*p* = 0.01). In addition, after adjusting for moderate physical activity and overweight status (model 2), the HDL-C level remained significantly associated with the cotinine level tertile (*p* = 0.04).

**Table 3:** A linear regression analysis of the association between urinary cotinine levels (tertiles) and metabolic syndrome components

Variable	Crude		Model 1^a^		Model 2^b^	
	*β* (95% CI)	*P* value	*β* (95% CI)	*P* value	*β* (95% CI)	*P* value
cMetS	0.45 (-0.02, 0.91)	0.062	0.38 (-0.10, 0.87)	0.123	0.21 (-0.21, 0.63)	0.325
MAP (mmHg)	0.59 (-0.80, 1.98)	0.403	0.41 (-1.02, 1.83)	0.574	0.28 (-1.13, 1.70)	0.694
SBP (mmHg)	0.75 (-1.10, 2.60)	0.425	0.50 (-1.39, 2.39)	0.603	0.18 (-1.67, 2.02)	0.851
DBP (mmHg)	0.51 (-0.85, 1.87)	0.461	0.36 (-1.04, 1.77)	0.613	0.34 (-1.07, 1.75)	0.639
BMI (kg/m^2^)	0.54 (0.02, 1.06)	0.043	0.49 (-0.05, 1.04)	0.074	-	-
FBG (mg/dL)	0.12 (-0.88, 1.12)	0.818	0.02 (-1.02, 1.05)	0.974	0.06 (-0.97, 1.09)	0.909
log TG^c^	0.00 (-0.07, 0.07)	0.983	0.00 (-0.07, 0.07)	0.926	-0.02 (-0.09, 0.05)	0.593
HDL-C (mg/dL)	-2.11 (-3.59, -0.63)	0.006	-1.93 (-3.45, -0.41)	0.013	-1.54 (-3.04, -0.05)	0.043

The range of urinary cotinine levels was divided into 1st tertile (≤ 0.356 ug/L), 2nd tertile (0.357–0.692 ug/L), 3rd tertile (> 0.692 ug/L).

95% CI, 95% confidence intervals; cMetS, continuous metabolic syndrome score; MAP, mean arterial pressure; SBP, systolic blood pressure; DBP, diastolic blood pressure; BMI, body mass index; FBG, fasting blood glucose; TG, triglyceride; HDL-C, high-density lipoprotein-cholesterol.

^a^ Model1 was adjusted for sex, father’s education level, father’s current drinking.

^b^ Model2 was adjusted for sex, father’s education level, father’s current drinking, moderate physical activity and overweight (≥ 85th percentile or not).

^c^ Log transformation was performed because it was not normally distributed.

## Discussion

SHS exposure in adolescents was associated with the components of MetS. In particular, the association between the HDL-C level and urinary cotinine level was maintained even after adjusting for various confounding factors.

A significant inverse relationship between SHS and HDL-C is in line with previous studies. In a meta-analysis of MetS according to SHS exposure [33], exposure in the younger age group (10–18 years old) showed a negative correlation with the HDL-C level. However, no significant association was found in the older age group (30–60 years). In addition, as a result of the Western Australian Pregnancy Cohort (Raine) Study, 17-year-old non-smoking girls exposed to SHS since birth had HDL-C levels on average 1.69 mg/dL lower compared with those not exposed to SHS [35]. Most of these studies investigated SHS exposure through questionnaires, but this study is meaningful in that it was the result of directly measuring SHS exposure and evaluating its relevance.

In our study, SHS and BMI showed a marginally significant relationship, but a previous study found a stronger association between SHS and BMI in adolescents (10–18 years old) than in children (0–10 years old) [33]. Another study reported that the risk of MetS increased by at least fourfold after exposure to SHS in overweight or at-risk adolescents [12].

In this study, there was no significant association between the urinary cotinine level and BP, but in previous studies, children and adolescents (8–19 years) exposed to smoking or SHS had a 35% higher risk of pre-hypertension compared with those who were not exposed. In particular, adolescents exposed only to SHS were found to have a 50% higher risk of BP increase [32, 36]. Therefore, exposure to smoking and SHS in adolescence may be a predictor of hypertension in adulthood. In adults, there is an association between SHS and metabolic markers [37, 38]. A recent study evaluating Korean adults also found that exposure to SHS was associated with increased hypertension [39]. Although smoking rates in adults and adolescents have decreased to 21.5% and 2.7%, respectively [5, 40], smoking remains a serious health problem. SHS in adulthood is a cardiovascular risk factor [41], and these results suggest that SHS and MetS are associated in childhood and adolescence.

Cotinine, one of the metabolites of nicotine in cigarettes, has a long half-life of 18–20 h and is recognized as an objective smoking indicator because it shows a stable concentration [42, 43]. Cotinine tests are useful for monitoring exposure to SHS [44] as well as determining whether adolescents smoke [45]. Cotinine concentrations have decreased significantly over the past decade, but exposure to SHS has not. We confirmed that 83.8% of the study subjects were exposed to SHS, suggesting that the self-reporting estimate significantly deviated from the cotinine level in urine [44]. In addition, regarding the survey and questionnaire response reliability, 10–40% of the respondents gave a false answer [45–47]. In other words, the self-reporting method for measuring exposure to SHS has the limitation of being subjective or inaccurate. In this study, the agreement between self-reported exposure to SHS and the level of cotinine in urine was low (*p* = 0.16). Measuring the urinary cotinine concentration as a biomarker of SHS could overcome subjective and inaccurate self-reporting and accurately reflect exposure to SHS from all places.

In this study, the association between the cMetS and the urinary cotinine level was insignificant. However, in the US National Health and Nutrition Examination Survey III (1988–1994), a linear relationship was found between the intensity of SHS exposure and MetS in 12–19-year-old adolescents [12]. From a public health perspective, it is important to assess the relationships of MetS and its components with exposure to SHS in adolescence to prevent cardiovascular disease risk factors in adulthood. The fact that smoking exposure and MetS are preventable [48, 49] has great implications for future public health. This study revealed associations of the cMetS and metabolic components with the cotinine level in adolescents, and based on the results of this study, an in-depth study is expected in the future.

Several limitations should be noted when interpreting the findings of this study. First, the subjects were from a specific hospital, which limits generalization of the study results. Second, as the urinary elimination half-life of cotinine is 18–20 h, SHS exposure may have been underestimated [42]. However, the maximum cotinine concentration in the subjects in this study was 4.50 µg/L, which is very low compared with the cut-off level (39.85 µg/L) for smoking in Korean adolescents [50]. In addition, the study subjects had general characteristics similar to those of Korean adolescents (e.g., prevalence of being overweight and obese), but the SHS exposure rate (this study 8.4%, KYRBS 25.4% in 2020) [5] and cotinine levels [51] were lower. Therefore, the findings of this study are meaningful, particularly because the association was shown even at very low cotinine levels and SHS exposure.

## Conclusion

The cotinine level in 13- to 15-year-old adolescents was determined from the results of the Ewha birth cohort study, and the associations between some MetS components and the urinary cotinine level were confirmed. This study was able to minimize the interference of bias and confounding factors via its prospective cohort design and adjustment for confounding factors. Due to a lack of studies on the association of MetS with the cotinine level in Korean adolescents, this study is considered to be of great significance. Since a relationship between MetS and the urinary cotinine level was found, efforts to reduce SHS exposure in adolescence are necessary.

## Data Availability

The cohort data are not freely available, but the Ewha Birth and Growth Study team welcomes collaborations with other researchers. For further information, contact Dr. Park (hpark@ewha.ac.kr).

## References

[1] Öberg M, Jaakkola MS, Prüss-Üstün et al. Annette, Peruga, Armando, Woodward, Alistair. Global estimate of the burden of disease from second-hand smoke. World Health Organization. 2000. https://apps.who.int/iris/handle/10665/44426. Accessed 21 Feb 2022.

[2] Makate M, Whetton S, Tait RJ (2020). Tobacco cost of illness studies: a systematic review. Nicotine Tob Res.

[3] Brody DJ, Lu Z, Tsai J, U.S. DEPARTMENT OF HEALTH AND HUMAN SERVICES, Centers for Disease Control and Prevention (2019). Secondhand smoke exposure among Nonsmoking Youth: United States, 2013–2016. NCHS(National Center for Health Statistics) data brief. August.

[4] Wang MP, Ho SY, Lo WS, Lam TH (2012). Smoking family, secondhand smoke exposure at home, and nicotine addiction among adolescent smokers. Addict Behav.

[5] Korean Disease Control and Prevention (KDCA)., The Korea Youth Risk Behavior Web-based Survey (KYRBS). 2020.

[6] Lai HK, Ho SY, Wang MP, Lam TH (2009). Secondhand smoke and respiratory symptoms among adolescent current smokers. Pediatrics.

[7] Omoloja A, Chand D, Greenbaum L, Wilson A, Bastian V, Ferris M (2011). Cigarette smoking and second-hand smoking exposure in adolescents with chronic kidney disease: a study from the Midwest Pediatric Nephrology Consortium. Nephrol Dial Transplant.

[8] U.S. Department of Health and Human Services. The Health Consequences of Involuntary Exposure to Tobacco Smoke: A Report of the Surgeon General. Atlanta: Coordinating Center for Health Promotion, National Center for Chronic Disease Prevention and Health Promotion, Office on Smoking and Health; 2006. Accessed 9 May 2022. U.S. Department of Health and Human Services, Centers for Disease Control and Prevention,. 20669524

[9] Kwon M, Lee J, Hyun SJ (2020). Effects of Seconhand smoke on Mental Health in Adolescents. J Korean Soc Sch Health.

[10] Chen R, Clifford A, Lang L, Anstey KJ (2013). Is exposure to secondhand smoke associated with cognitive parameters of children and adolescents? -a systematic literature review. Ann Epidemiol.

[11] Schwartz J, Bottorff JL, Richardson CG. Secondhand smoke exposure, restless sleep, and sleep duration in adolescents.Sleep Disorders. 2014:2014:374732. 10.1155/2014/374732 PMC3997916 24808961

[12] Weitzman M, Cook S, Auinger P, Florin TA, Daniels S, NguyenM (2005). Tobacco smoke exposure is associated with the metabolic syndrome in adolescents. Circulation.

[13] Isomaa B, Almgren P, Tuomi T, Forsen B, Lahti K, Nissen M (2001). Cardiovascular morbidity and mortality associated with the metabolic syndrome. Diabetes Care.

[14] Chae J, Seo MY, Kim SH, Park MJ (2021). Trend and Risk factors of metabolic syndrome among korean adolescents, 2007 to 2018. Diabetes Metab J.

[15] Morrison JA, Friedman LA, Gray-McGuire C (2007). Metabolic syndrome in childhood predicts adult cardiovascular disease 25 years later: the Princeton lipid Research Clinics follow-up study. Pediatrics.

[16] Morrison JA, Friedman LA, Wang P, Glueck CJ (2008). Metabolic syndrome in childhood predicts adult metabolic syndrome and type 2 diabetes mellitus 25 to 30 years later. J Pediatr.

[17] Thangiah N, Chinna K, Su TT, Jalaludin MY, Al-Sadat N, Majid HA. Clustering and Tracking the Stability of Biological CVD Risk Factors in Adolescents: The Malaysian Health and Adolescents Longitudinal Research Team Study (MyHeARTs).Frontiers in public health.2020;17;8:69. 10.3389/fpubh.2020.00069 PMC7090141 32257989

[18] Lee HA, Park B, Min J, Choi EJ, Kim UJ, Park HJ (2021). Cohort Profile: the Ewha Birth and Growth Study. Epidemiol Health.

[19] Habler K, Paal M, Vogeser M. Isotope dilution-LC-MS/MS method for quantification of the urinary cotinine-to-creatinine ratio. Clin Chem Lab Med. 2020 Mar;25(9):1469–76. 10.1515/cclm-2020-0177 32229659

[20] National Institute of Environmental Research (NIER). QA/QC Handbook for the Environmental Pollutants. Analysis and Sampling Techniques. 2011. Accessed 30 May 2021.

[21] Goodman E, Daniels SR, Meigs JB, Dolan LM. Instability in the diagnosis of metabolic syndrome in adolescents. Circulation. 2007 May 1;115(17):2316–22. 10.1161/CIRCULATIONAHA.106.669994 PMC2626638 17420347

[22] Gurka MJ, Ice CL, Sun SS (2012). A confirmatory factor analysis of the metabolic syndrome in adolescents: an examination of sex and racial/ethnic differences. Cardiovasc Diabetol.

[23] Reinehr T, de Sousa G, Toschke AM, Andler W (2007). Comparison of metabolic syndrome prevalence using eight different definitions: a critical approach. Arch Dis Child.

[24] World Health Organization. (1999). Definition, diagnosis and classification of diabetes mellitus and its complications: report of a WHO consultation. Part 1, Diagnosis and classification of diabetes mellitus. World Health Organization. https://apps.who.int/iris/handle/10665/66040.

[25] Dania AH, Vandana R (2017). Metabolic syndrome in children and adolescents. Transl Pediatr.

[26] Wijndaele K, Beunen G, Duvigneaud N (2006). A continuous metabolic syndrome risk score: utility for epidemiological analyses. Diabetes Care.

[27] Okosun IS, Lyn R, Davis-Smith M, Eriksen M, Seale P (2010). Validity of a continuous metabolic risk score as an index for modeling metabolic syndrome in adolescents. Ann Epidemiol.

[28] Heshmat R, Heidari M, Ejtahed H-S, Motlagh ME, Mahdavi-Gorab A (2017). Validity of a continuous metabolic syndrome score as an index for modeling metabolic syndrome in children and adolescents: the CASPIAN-V study. Diabetol Metab Syndr.

[29] Kelly AS, Steinberger J, Jacobs DR, Hong CP, Moran A, Sinaiko AR (2011). Predicting cardiovascular risk in young adulthood from the metabolic syndrome, its component risk factors, and a cluster score in childhood. Pediatr Obes.

[30] Gurka MJ, Filipp SL, Musani SK (2018). Use of BMI as the marker of adiposity in a metabolic syndrome severity score: derivation and validation in predicting long-term disease outcomes. Metabolism.

[31] Melgarejo JD, Yang W-Y, Thijs L (2021). Association of Fatal and Nonfatal Cardiovascular outcomes with 24-Hour Mean arterial pressure. Hypertension.

[32] Rebecca VL, Brathwaite KE, Sarathy H (2021). Analysis of active and Passive Tobacco Exposures and blood pressure in US children and adolescents. JAMA Netw Open.

[33] Chen H-J, Li G-L, Sun A, Peng D-S (2019). Age differences in the relationship between secondhand smoke exposure and risk of metabolic syndrome: a Meta-analysis. Int J Environ Res Public Health.

[34] Korea Center for Disease Control and Prevention (KDCA). Korean children and adolescents 2017 growth chart commentary. Available at https://knhanes.kdca.go.kr/knhanes/sub08/sub08_01.do. Accessed on May 8, 2022.

[35] Le-Ha C, Beilin LJ, Burrows S, Huang R-C (2013). Gender difference in the relationship between Passive Smoking exposure and HDL-Cholesterol levels in late adolescence. JCEM.

[36] Shelley H, Liu B, Liu, Alison PS, Jeffrey S, Karen MW (2020). Secondhand smoke exposure and higher blood pressure in children and adolescents participating in NHANES. Prev Med.

[37] Kim JH, Kim BJ, Hyun YY, Kang JH (2020). Association between secondhand smoke exposure and metabolic syndrome in 118,609 korean never smokers verified by self-reported questionnaire and urine cotinine. Endocrinol Metabolism.

[38] U.S. Department of Health and Human Services (2010). A report of the Surgeon General: how Tobacco smoke causes Disease: what it means to you.

[39] Kim BJ, Kang JG, Kim JH, Seo DC, Sung KC, Kim BS (2019). Association between secondhand smoke exposure and hypertension in 106,268 korean self-reported never-smokers verified by cotinine. J Clin Med.

[40] Korean Disease Control and Prevention (KDCA)., 2019 Korea National Health&Nurtition Examination Survey. 2020.

[41] He J, Vupputuri S, Allen K, Prerost MR, Hughes J, Whelton PK (1999). Passive smoking and the risk of coronary heart disease- a meta-analysis of epidemiologic studies. N Engl J Med.

[42] Jarvis MJ, Tunstall-Pedoe H, Feyerabend C, Vesey C, Saloojee Y (1987). Comparison of tests used to distinguish smokers from nonsmokers. Am J Public Health.

[43] Lerman C, Orleans CT, Engstrom PF (1993). Biological markers in smoking cessation treatment. Semin Oncol.

[44] Park MB, Sim B (2020). Differences in environmental Tobacco smoke exposure between self-reporting and cotinine test: the application of biomarkers. Health Policy and Management.

[45] Sillett RW, Wilson MB, Malcolm RE, Ball KP (1978). Deception among smokers. Br Med J.

[46] Wilcox RG, Hughes J, Roland J (1979). Verification of smoking history in patients after infarction using urinary nicotine and cotinine measurements. Br Med J.

[47] Apseloff G, Ashton HM, Friedman H, Gerber N (1994). The importance of measuring cotinine levels to identify smokers in clinical trials. Clin Pharmacol Ther.

[48] Kim J, Park D, Kang B, Choi H (2017). The influence of Passive Smoking in Home on Smoking in Korean Adolescent: Korea Youth Risk Behavior web-based survey (2014). Korean J Fam Pract.

[49] Azadbakht L, Mirmiran P, Esmaillzadeh A, Azizi T, Azizi F (2005). Beneficial effects of a Dietary Approaches to stop hypertension eating plan on features of the metabolic syndrome. Diabetes Care.

[50] Jung S, Park S (2021). Determination of urinary cotinine cut-off point for discriminating smokers and non-smokers among adolescents: the third cycle of the Korean National Environmental Health Survey (2015 ~ 2017). J Environ Health Sci.

[51] Park D, Kim H, Lee S (2016). Trends in Secondhand smoke exposure among nonsmokers in the Korean Population: the 2008–2011 korean National Health and Nutrition Examination Survey. Korean J Fam Pract.

